# Jejunal Amyloidoma - a rare cause of gastrointestinal bleeding

**DOI:** 10.1186/1757-1626-2-9100

**Published:** 2009-11-27

**Authors:** Tolutope Oyasiji, Steven Yood

**Affiliations:** 1 Department of Surgery, Hospital of Saint Raphael, 1450 Chapel Street, New Haven, CT 06511, USA

## Abstract

We report a case of localized amyloid tumor of the jejunum which presented with abdominal pain and gastrointestinal bleeding. We reviewed the pathophysiologic process that precipitates bleeding in this rare tumor. We also examined the documented radiologic and endoscopic features of amyloidosis of the small bowel in the light of our reported case. All with a view to add to the growing evidence on this rare tumor which will facilitate accurate diagnosis and management.

## Case Report

A 59-year old Caucasian female admitted to medical service for syncope and anemia. Initial evaluation revealed left lower quadrant abdominal pain and passage of maroon-colour stool which had been present for two days prior to the admission.

She has a past medical history which is significant for undefined hypercoagulable disorder characterized by 2 episodes of pulmonary embolism, hypothyroidism, Riedel's thyroiditis for which she had thyroidectomy, depression and obsessive-compulsive disorder. She was on the following medications- Coumadin, Synthroid, and Klonopin.

Physical examination revealed a middle-aged woman who was afebrile, tachycardic with heart rate of 136 beats per minute and orthostatic hypotension [supine blood pressure - 113/85 mmHg and 80/60 mmHg in sitting position].

Pulmonary examination was normal

Abdominal examination revealed a mildly but diffusely tender abdomen with grossly heme positive stool on rectal examination.

Initial laboratory work-up showed the following:

Complete blood count- white cell count 17,300/mm3, hematocrit 27.6%, platelet 167,000/mm3

Coagulation profile- PT 51.4 seconds, PTT 46.5 seconds, INR 15.3

Biochemical profile was normal

**Figure 1:**
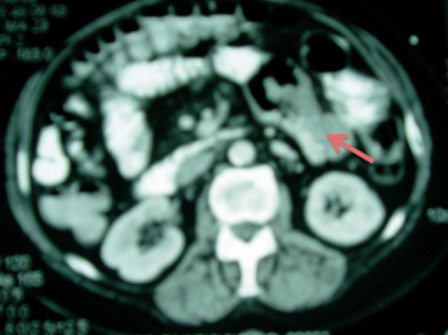
**CT Abdomen showing the mass in the proximal small bowel**.

In view of abdominal pain and lower gastrointestinal bleeding, a computerized tomography scan of the abdomen and pelvis was ordered which revealed a mass within the small bowel associated with thickened small bowel wall and mesenteric lymphadenopathy [fig 1].

**Figure 2:**
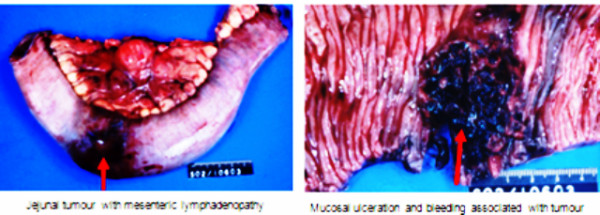
**Resected segment of jejunum containing the amyloidoma**.

She had the anticoagulation reversed with fresh frozen plasma and her INR quickly corrected to 1.4. She also had 2 units of blood transfused which raised the hematocrit to 28.1. She underwent laparotomy about 36 hours into the admission. Operative findings include a 5 cm mass in the mid jejunum with associated mesenteric lymphadenopathy [fig 2]. The affected jejunal segment was resected and a side-to-side anastomosis was done. She had an uneventful post-operative course and was discharged home on post-operative day 5.

**Figure 3:**
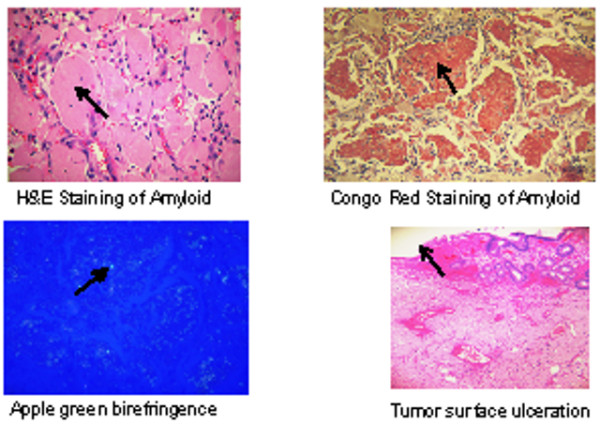
**Histology slides with H&E stain, Congo Red stain, apple-green birefringence under polarized light and slide showing mucosal ulceration**.

Pathology showed amyloidoma associated with ulceration and extensive recent and organizing hemorrhage. Congo red staining of the tumor was done and apple-green birefringence under polarized light was demonstrated [fig 3]. Adjacent bowel proximally and distally to the tumor did not stain with Congo red.

## Discussion

Amyloidosis often present in the systemic form while localized amyloidosis constitutes 10-20% of cases [1]. The involvement of the gastrointestinal tract in amyloidosis is well documented. Some authors have documented as high as 98% incidence of gastrointestinal tract involvement in systemic amyloidosis [2,3]. Localized gastrointestinal amyloidosis is uncommon, with few cases reported in the literature. Literature search also revealed that the proximal small bowel is the most common part of gastrointestinal tract affected [4-6]. Gastrointestinal amyloidosis can present clinically as abdominal pain, diarrhea, pseudo obstruction, malabsorption, bleeding and perforation [7].

This is a case of localized gastrointestinal amyloidosis presenting as a bleeding tumor which clinically manifested as abdominal pain and lower gastrointestinal bleeding. We report this case with the aim to review the pathologic process responsible for amyloidosis causing gastrointestinal bleeding. The radiologic and endoscopic features of small bowel amyloidosis are also examined.

Various concepts have been described in an attempt to explain why amyloidosis produces gastrointestinal hemorrhage. Amyloidosis angiopathy is described as the process by which amyloid deposition in the wall of the intestinal blood vessels causes fragility of the vessel wall and predisposes to bleeding [8]. Another concept holds that progressive ischemia due to compromised vessel lumen and blood supply as a result of intramural deposition of amyloid on vessel wall results in necrotic ulceration and consequent bleeding [9]. Disturbances in coagulation can also account for bleeding from amyloidosis [3]. Our reported case was a patient on Coumadin treatment for an undefined hypercoagulable disorder. At the time of presentation with the bleeding jejunal amyloidoma, the INR was supratherapeutic at a level of 15.3. In another reported case with bleeding, the patient was on Coumadin for anticoagulation prior to electric cardioversion for atrial fibrillation [9].

Computerized tomography scan of the abdomen in this patient revealed a soft-tissue density tumor in the proximal small bowel. Differential diagnoses considered by the radiologist are lymphoma, intussusception and hematoma. Other reported cases of amyloidosis in the small bowel have documented difficulty with radiologic diagnosis with radiologists opting for differential diagnoses like metastatic tumors, small bowel adenocarcinoma and lymphoma. This is not unexpected as primary small bowel tumors are uncommon and amyloid tumors of the small bowel are rare [9]. Characterization of the radiographic patterns in small bowel amyloidosis has been done largely through small bowel series. Documented patterns include thickening of the folds, jejunization of ileum, impaired intestinal motor activity, innumerable fine granular elevations, polypoid protrusions and tumor-like lesions [3]. Unfortunately, the clinical scenario in our reported case with worsening abdominal pain and ongoing bleeding did not leave room for doing a small bowel series before surgical resection. By and large, it is becoming increasingly important to consider the possibility of amyloidosis in the setting of small bowel tumor presenting either with abdominal pain, bleeding or obstruction.

Endoscopic features like fine, granular appearance, erosions, mucosal friability, thickening of the valvulae conniventes, multiple polypoid protrusions and shallow ulcers were reported by Tada et al [3,10]. Our patient did not have a small bowel endoscopy done. However, macroscopic findings of the resected specimen revealed a 4 cmx4 cm friable, ulcerated and fungating tumor. Mucosa adjacent to the tumor was also granular. The presence of ulceration, friability of the tumor and granular surrounding mucosa are consistent with the described endoscopic features.

This reported case is an important addition to the growing body of evidence on gastrointestinal amyloidosis. In view of the rarity of focal amyloidosis of the small bowel and the broad range of possible clinical presentations, it becomes judicious to closely review every diagnosed case to establish consistent, common findings that will make future diagnosis of the condition easier and more accurate.

## Consent

Written informed consent was obtained from the patient for publication of this case report and accompanying images. A copy of the written consent is available for review by the Editor-in-Chief of this journal.

## Competing interests

The authors declare that they have no competing interests.

## Authors' contributions

OT and YS contributed to the review of the patient's chart, collation of the data, images and information needed for writing the case report. Both authors also contributed to writing the case report.
